# A Gestational Pectin Diet Could Improve the Health of Multiparous Sows by Modulating the Gut Microbiota and Cytokine Level during Late Pregnancy

**DOI:** 10.3390/ani14111559

**Published:** 2024-05-24

**Authors:** Jie Zheng, Shuang Li, Jiaqi He, Hao Liu, Yingyan Huang, Xuemei Jiang, Xilun Zhao, Jian Li, Bin Feng, Lianqiang Che, Zhengfeng Fang, Shengyu Xu, Yan Lin, Lun Hua, Yong Zhuo, De Wu

**Affiliations:** 1 Key Laboratory for Animal Disease-Resistance Nutrition, China Ministry of Education, Institute of Animal Nutrition, Sichuan Agricultural University, No. 211, Huimin Road, Chengdu 611130, China; 2 College of Bioengineering, Sichuan University of Science & Engineering, Yibin 644000, China

**Keywords:** pectin, sow, microbiota, SCFAs, cytokines

## Abstract

**Simple Summary:**

Maintaining the health of sows in pig production poses a persistent and significant challenge that directly impacts sow reproductive performance as well as producer economics. Pectin, a dietary fiber with immense potential, was investigated in this study for its potential to enhance the health of pregnant sows. Throughout gestation, we supplemented the basic diet with 3% of low-ester pectin to examine its potential effects in pregnant sows. We collected and analyzed serum and fecal samples from the sows to evaluate their fecal microbiota, serum metabolites, and cytokines, aiming to provide fundamental data for pectin utilization in animal production.

**Abstract:**

This study aimed to investigate the effects of the dietary fiber pectin on the gut microbiota and health of parturient sows. A total of 30 parity 5–7, multiparous gestation sows (Large White × Landrace) were randomly assigned to two treatment groups after mating: Con (control, basic diet) and Pec (pectin, 3%). The sows received the two diets during gestation, and all sows were fed the same standard basic diet during lactation. The results of β-diversity showed that the composition of the gut microbiota was different in the Con and Pec groups. Compared with the sows in the Con group, the Pec sows showed a higher abundance of the gut bacteria *Clostridium* and *Romboutsia* and a lower abundance of harmful bacteria (*Micrococcaceae*, *Coriobacteriaceae*, *Dorea*, *Actinomyces*). On the other hand, the SCFA plasma concentration was increased in the Pec group, while pro-inflammatory cytokine (IL-6, IL-1β, and TNF-α) concentrations were decreased. In conclusion, the soluble dietary fiber pectin could improve the reproductive performance and health of sows by increasing the abundance of some commensal bacteria enhancing the metabolite SCFA levels and reducing the pro-inflammatory cytokine plasma levels.

## 1. Introduction

Gestation is a crucial stage in the reproductive cycle of sows. However, sows undergo drastic changes in hormone secretion, immune response, and physiological and metabolic characteristics during the whole gestation [1]. Especially in the third trimester, sows could suffer from metabolic syndrome which includes insulin resistance, intestinal inflammation, and elevated systemic inflammation levels [1,2,3]. The presence of moderate insulin resistance, inflammation, and free radicals in a normal pregnancy plays a beneficial role in maintaining pregnancy [4]. The associated risks are evident. An evolutionary explanation proposes that the maternal immune reaction assists in aborting unhealthy pregnancies while allowing healthy ones to continue [5]. However, the excessive occurrence of metabolic syndrome results in cellular and tissue damage in the sow [6], thereby giving rise to a spectrum of pregnancy disorders including preterm birth and pre-eclampsia [7,8,9]. For instance, chorioamniotic inflammation is more prevalent among obese pregnant women and has been linked to the premature rupture of membranes, preterm birth, increased neonatal mortality rates, as well as an elevated incidence of cerebral palsy among the survivors [8,9]. Cheng et al. (2018) suggested that systemic low-grade inflammation and insulin resistance in perinatal sows reduced the number and litter weight of the piglets born alive and affected the intestinal health of piglets [1,10]. Li et al. (2023) suggested that a high inflammation level would be harmful to sows’ lactation performance [11]. Emerging data in mouse and sow experiments indicated that dysbiosis of the gut microbiota may induce an increase in gut permeability [1,12]. This observation suggests that gut microbiota alterations may directly influence maternal pregnancy-associated metabolic changes [13,14,15]. Microbiological changes may be closely related to sow health during gestation.

In previous studies, dietary fiber (DF) has shown many benefits for pig production, such as increased satiety and reduced occurrence of stereotypical behavior in pregnant sows, improving sow welfare [16]. DF could improve the reproductive performance of sows [17], the piglet survival rate, and the performance of sows during lactation [18]. Pectin is one of the most common sources of dietary fiber for human and animal nutrition [19]. Pectin is a family of complex polysaccharides, primarily composed of repeating units of galacturonic acid connected by α-(1-4) glycosidic linkages to form linear polymers [20]. Pectin is found in all plants’ cell walls, but it is most abundant in citrus fruits [21]. Previous in vitro and in vivo studies demonstrated citrus pectin’s antioxidative [22], anti-diabetic [23], anti-inflammatory [24], and other immunomodulatory activities [25]. Many functional properties of pectin are dependent on the degree of esterification as well as on other structural parameters. Recent reports demonstrated the potential of citrus pectin to attenuate endotoxin shock via the suppression of Toll-like receptor signaling in Payer´s patch myeloid cells, both stimulating and suppressing the immune response [26]. A low degree of methyl esterification in citrus pectin used as a source of dietary fiber could block immune receptors in human dendritic cells and murine macrophages by inhibiting the Toll-like receptor 1 (TLR1) and Toll-like receptor 2 (TLR2) proinflammatory pathways [24]. The addition of fermentable fiber such as pectin to pig diets may stimulate the growth and metabolic activity of beneficial bacterial species in the gut [27,28]. Pectin could favor the adhesion of probiotic *Lactobacillus* strains to epithelial cells [29]. In vitro fermentation with pectins stimulated various beneficial bacteria, including *Bacteroides*, *Bifidobacteria*, *Clostridiales*, *Prevotella*, and *Lactobacilli* [30,31]. In addition, the genera Bacteroides and Prevotella are the primary pectin degraders, as they possess genes coding for carbohydrate-active enzymes (CAZymes) within their polysaccharide utilization loci (PUL) [32]. These bacteria use lyases, methylesterases, and acetylases to break down pectin molecules [33]. The microorganisms residing in the gut of animals possess the capability to synthesize a diverse array of enzymes to ferment and metabolize pectin via polysaccharide utilization sites and produce beneficial metabolites, such as SCFAs. The involvement of SCFAs can be observed in a diverse range of metabolic pathways within the body [34,35]. Furthermore, recent studies indicated that citrus pectin blocked Gal-3 activity by reducing macrophage activity, proinflammatory cytokine expression, and apoptosis, lowering inflammatory marker levels and improving animal health [36]. In recent years, many studies, through in vitro fermentation tests and mouse tests, have reported significant effects of pectin on animal metabolism and the gut microbiota. The main effects on animals involve antioxidant, anti-inflammatory, and other benefits. However, reports relating the effects of pectin on sows are limited. Therefore, the purpose of our study was to explore whether pectin fiber can affect the gut microbiota and promote the beneficial production of SCFAs in pregnant sows, reduce the occurrence of excessive inflammation during late pregnancy, and improve the reproductive performance of sows.

## 2. Materials and Methods

This study was performed at the Research Farm of the Animal Nutrition Institute, Sichuan Agricultural University, Ya’an, China. The present experiments complied with the guidelines from the Animal Care and Use Committee of Sichuan Agricultural University and followed the current laws of animal protection.

### 2.1. Animals and Diets

A total of thirty multiparous sows (Large White × Landrace) with parity 5–7 and a similar body weight (BW) (228.16 ± 6.16 kg) and backfat (BF) thickness (12.88 ± 0.26 mm) were used in this study. The sows were artificially inseminated with semen from a pool of Duroc boars on the day of estrus and then 12 and 24 h later. They were housed in individual gestation stalls (2.13 × 0.61 m). After artificial insemination, the sows were weighed and randomly allocated to 2 treatments with pectin (Pec, 3%) and control (Con) diets. Each treatment group contained 15 replicated pens, each with one sow. All diets met the nutrient requirements of gestating sows as recommended by the NRC (2012) (Table 1). The sows were fed the experimental diets from day 0 (first artificial insemination) of gestation until farrowing; the feeding quantity of the two group sows at each stage of gestation is shown in Table 2. The average feed intake and daily nutrient intake of the sows during pregnancy are shown in Table 3. All sows were fed twice daily at 08:00 and 15:30. The sows were transferred to farrowing stalls and then individually placed in waiting areas for delivery on day 110 of gestation. 

### 2.2. Animal Sample Collection

#### 2.2.1. Sow Blood Samples 

Fasted blood samples were collected from the auricular veins of each sow before their morning meal on day 110 of gestation. The blood samples (10 mL) were collected into heparinized tubes, maintained at a temperature of 4 °C for 30 min, and subsequently centrifuged for 15 min at a speed of 3000× *g* at 4 °C. Plasma samples were then harvested and stored at −20 °C until the analysis of plasma biochemical indexes, cytokines, SCFAs, and intestinal permeability indexes was conducted on day 110 during gestation.

#### 2.2.2. Sow Fecal Samples 

Fresh fecal samples were collected from the rectum in the morning on day 110 of gestation. After extraction, the inner part of the feces was transferred into sterile cryopreservation tubes and immediately stored at −80 °C. The duplicate fecal samples were individually analyzed for their microbial composition.

### 2.3. Performance Measurement

BF thickness was measured at 65 mm to the left side of the dorsal mid-line at the level of the last rib (P2) using B-mode ultrasonography (LN-9300A, Liaoning Caresono Technology Co., Ltd., Dandong, Liaoning Province, China) and recording on days 1, 30, 60, 90, and 110 of gestation. The body weight of the sows was measured on days 1, 30, 60, 90, and 110 of gestation.

### 2.4. Feedstuff Samples

The dietary fiber pectin used in the test was a soluble dietary fiber (SDF) extracted from citrus. This test showed that the esterification degree (DE) of pectin was 27%, and the purity was 87% (it mostly consisted of soluble dietary fiber), indicating that it was a high-purity low-ester pectin; it was provided by Chengdu Tupaite Technology Co., Ltd, Chengdu, China.

### 2.5. Chemical Analyses

#### 2.5.1. Analysis of Plasma Inflammatory Cytokines and Intestinal Permeability Index

Commercial ELISA kits (Jiangsu Meimian Industrial Co., Ltd., Yancheng, China) were used for determining the plasma concentrations of IL-1β, IL-6, IL-10, TNF-α, IFN-γ, LCN-2, zonulin, ZO-1, and occludin in the sows.

#### 2.5.2. Microbiological Analysis of Sow Feces

Microbial community genomic DNA was extracted from the fecal samples collected on day 110 of gestation (the sample size was 9 and 9 for the Pec and Con groups, respectively) using the E.Z.N.A. soil DNA kit (Omega Bio-tek, Norcross, GA, USA) according to the manufacturer’s instructions. DNA concentration and purity were determined with a NanoDrop 2000 UV-vis spectrophotometer (Thermo Scientific, Wilmington, NC, USA). The hypervariable region V3-V4 of the bacterial 16S rRNA gene was amplified with the primer pairs 338F (5′-ACTCCTACGGGAGGCAGCAG-3′) and 806R (5′-GGACTACHVGGGTWTCTAAT-3′) by an ABI GeneAmp 9700 PCR thermocycler (ABI, Foster city, CA, USA) and sequenced on an Illumina MiSeq PE300 platformvaSeq PE250 platform (Illumina, San Diego, CA, USA) according to standard protocols by Majorbio Bio-Pharm Technology Co. Ltd. (Shanghai, China), which generated 300 bp single-end reads. 

#### 2.5.3. Analysis of Plasma SCFA Levels in the Sows

The concentrations of SCFAs, including acetic acid, propionic acid, and butyric acid, in the plasma samples, were determined by CP-3800 gas chromatography (Varian, Inc., Palo Alto, CA, USA) according to an improved method [37]. Approximately 400 μL of the plasma samples was thawed and diluted, and then the supernatants were mixed with 50 μL of a 25% metaphosphoric acid solution and 4 μL of 21 mmol/L crotonic acid, and the mixed solution was placed at 4 °C for 30 min before centrifuging at 12,000× *g* for 10 min, Then, 100 μL of the supernatant was mixed with 100 μL of methanol and centrifuged at 20,000× *g* for 15 min. After completion, sample determination was performed.

### 2.6. Statistical Analyses

#### 2.6.1. Reproductive Performance Data, Plasma SCFA and Cytokines Concentration Data Analysis

The sows were considered as experimental units. Before the parametric analysis, descriptive statistics were performed to check the normality and homogeneity of variance. The total number of piglets born, piglets born alive, stillborn, mummified fetuses, and piglets born alive weighing <800 g was analyzed using the GLIMMIX procedure with the Poisson distribution. The BW and BF of the sows during gestation were determined using the SAS MIXED procedure for repeated measurements. The plasma SCFA and cytokines concentrations and the intestinal permeability index were analyzed using the independent samples t-test procedure of the SAS statistical package (version 9.4; SAS Institute, Inc. Cary, NC, USA) in a completely randomized design. The data are shown as least squares mean with standard errors for each treatment, unless otherwise stated. *p* < 0.05 indicated a significant difference, while 0.05 < *p* < 0.1 indicated a trend. 

#### 2.6.2. Microbiota Data Analysis

The bray_curtis method was used to draw PRINCIPAL coordinate analysis (PCoA) plots to visualize differences in the composition of the bacterial community among the samples. Nonparametric analyses (analysis of similarity, ANOSIM) for multivariate data were performed using the “WGCNA”, “stats”, and “ggplot2” packages in R (Version 2.15.3) for bacterial community structure comparison. The differences in relative abundance of bacteria between the groups were analyzed by Wilcoxon rank-sum test bar plot analysis.

## 3. Results 

### 3.1. Body Weight and Backfat Thickness Changes in the Sows

**Figure 1:**
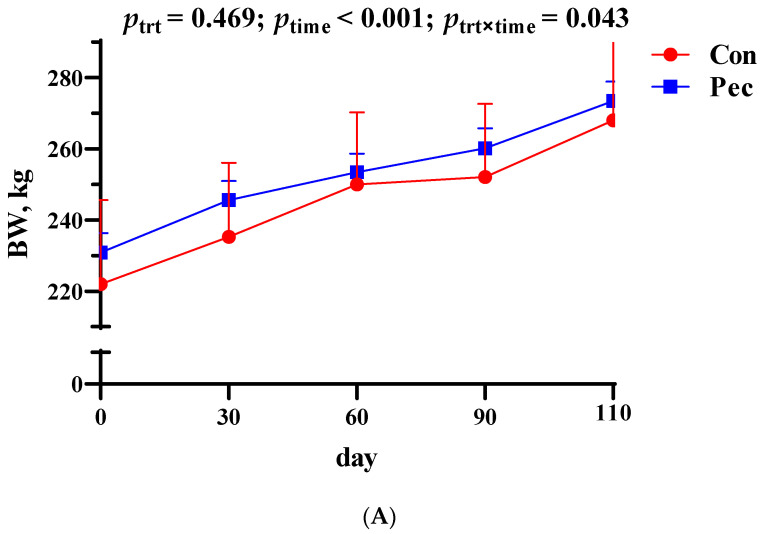

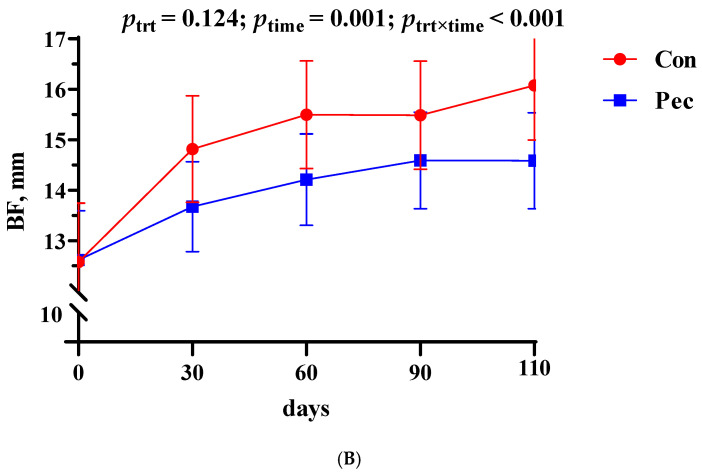
Effects in Pec and Con groups during gestation. (**A**) Body weight (BW) and (**B**) backfat (BF) thickness changes during gestation. Pec, pectin diet during gestation; Con, control diet during gestation. Data are expressed as least squares mean ± SE. Sows were regarded as experimental units, (**A**,**B**): Pec, *n* =15; Con, *n* = 15.

All sows in each group consumed the daily feed completely during the whole gestation period, and no feed residue was recorded. The changes in body weight (BW) and body fat (BF) thickness in the sows during pregnancy are shown in Figure 1. There was no change in body weight or BF thickness in the sows receiving the two treatments at various stages during gestation (*p* > 0.05). 

### 3.2. Production Performance of the Sows

As regards sows’ reproductive performance, we found that for the Pec sows, the number of mummified fetuses tended to decrease (*p* < 0.10), and the litter weight at birth tended to increase (*p* = 0.058) compared with those measured for the Con group sows. During lactation, BW and BF thickness as well as feed intake in in both groups did not change statistically (Table 4).

### 3.3. Microbiological Analysis of the Sow Fecal Samples

#### 3.3.1. Differences in Sow Microbiota Depending on the Treatment

**Figure 2:**
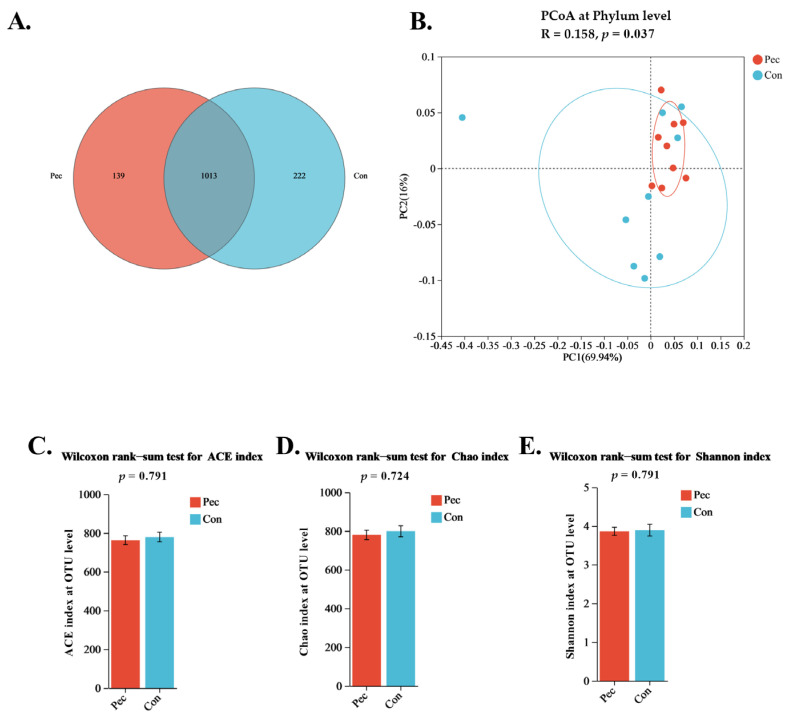
Effects in Pec and Con sows on day 110 of gestation on the gut microbiota structure of the sows. (**A**) Veen diagram of the sow fecal microbiota comparing Pec and Con sows at the OTU level; (**B**) principal coordinate analysis of the gut microbiota communities of Pec and Con sows at the phylum level; (**C**) ACE**;** (**D**) Chao, and (**E**) Shannon indexes of the gut microbiota on day 110 of gestation. Pec, pectin, *n* = 9; Con, control, *n* = 9.

As the Venn diagram shows (Figure 2A), the sows in the two groups presented common and special operational taxonomic units (OTUs). In total, 1013 common OTUs were found in the two groups, and 139 (~10.12% of the total OTUs) and 222 (~16.16% of the total OTUs) unique OTUs were identified, respectively, in the fecal microflora of the Pec and Con sows. For β-diversity, the distribution of the microbiota community in the two groups of sows showed obvious clustering along the principal coordinate, indicating that pectin, as a dietary fiber source added during gestation, can change the diversity of the gut microbial community in sows (Figure 2B). As shown in Figure 2C–E (ACE, Chao, and Shannon indexes), there was no significant difference in the α-diversity index between the two groups (*p* > 0.05). 

#### 3.3.2. Changes in Relative Abundance at the Phylum, Family, and Genus Levels after the Two Treatments

**Figure 3:**
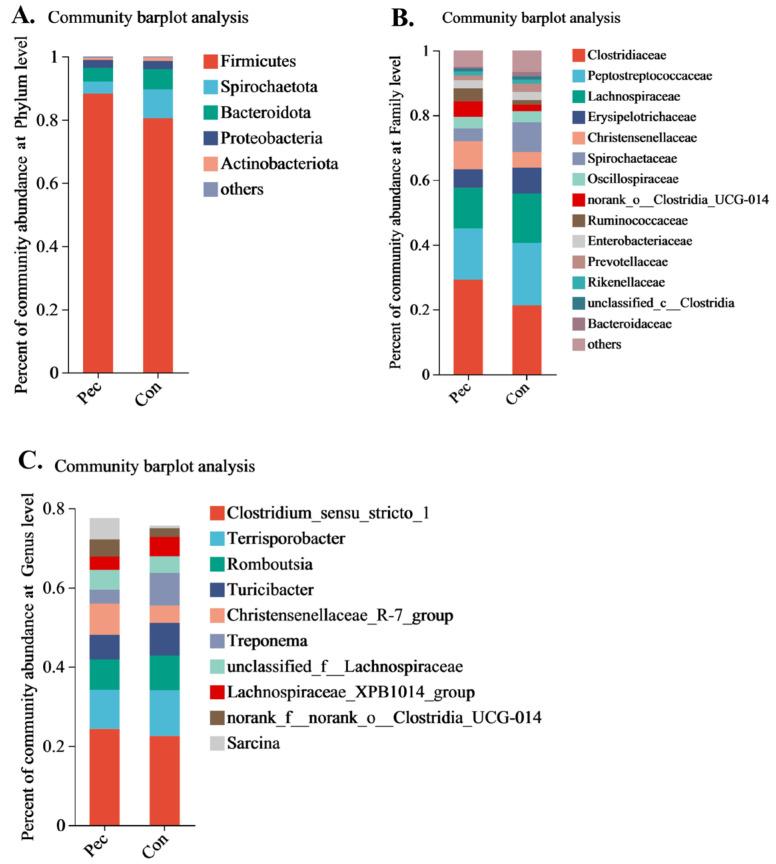
The relative abundance of the gut microbiota at different levels in Pec and Con sows on day 110 of gestation. (**A**) Phylum level; (**B**) family level; (**C**) genus level. Pec, pectin, *n* = 9; Con, control, *n* = 9.

**Figure 4:**
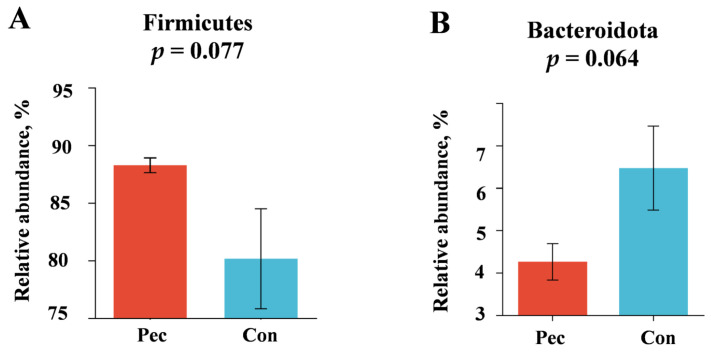
Effects in Pec and Con sows on the gut microbiota at the phylum level on day 110 of gestation. (**A**) *Firmicutes*; (**B**) *Bacteroidota*. Pec, pectin, *n* = 9; Con, control, *n* = 9.

**Figure 5:**
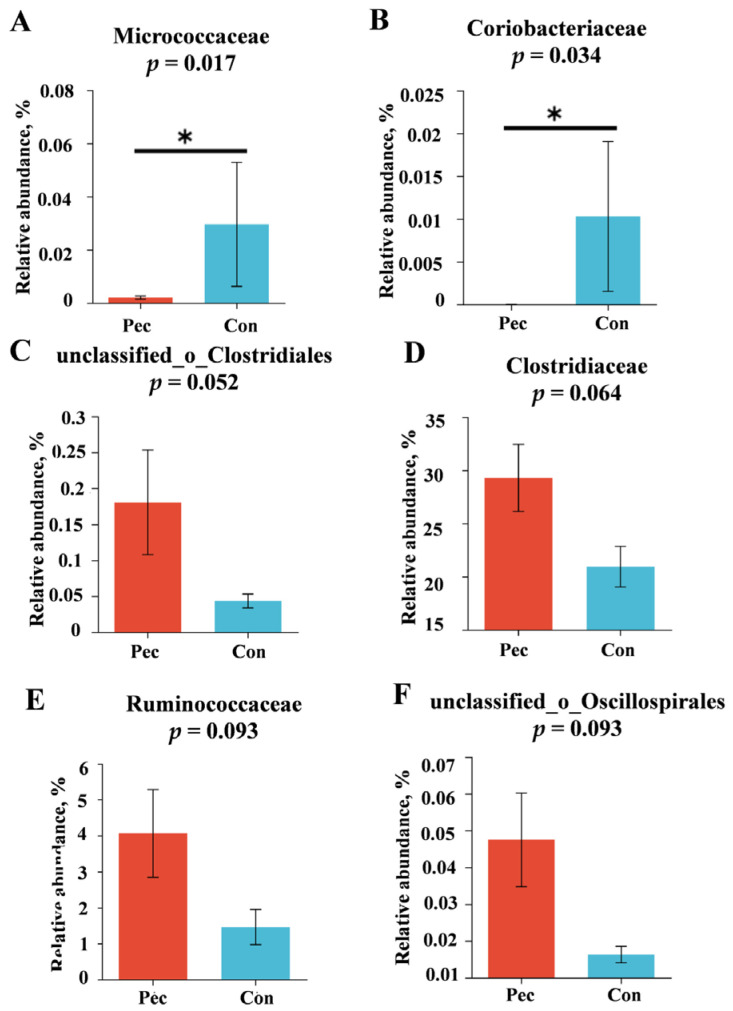
Effects in Pec and Con sows on the gut microbiota at the family level on day 110 of gestation. (**A**) *Micrococcaceae*; (**B**) *Coriobacteriaceae*; (**C**) *unclassified_o_Clostridiales*; (**D**) *Clostridiaceae*; (**E**) *Ruminococcaceae*; (**F**) *unclassified_o_Oscillispirales*. Pec, pectin, *n* = 9; Con, control, *n* = 9. * indicates *p* < 0.05.

**Figure 6:**
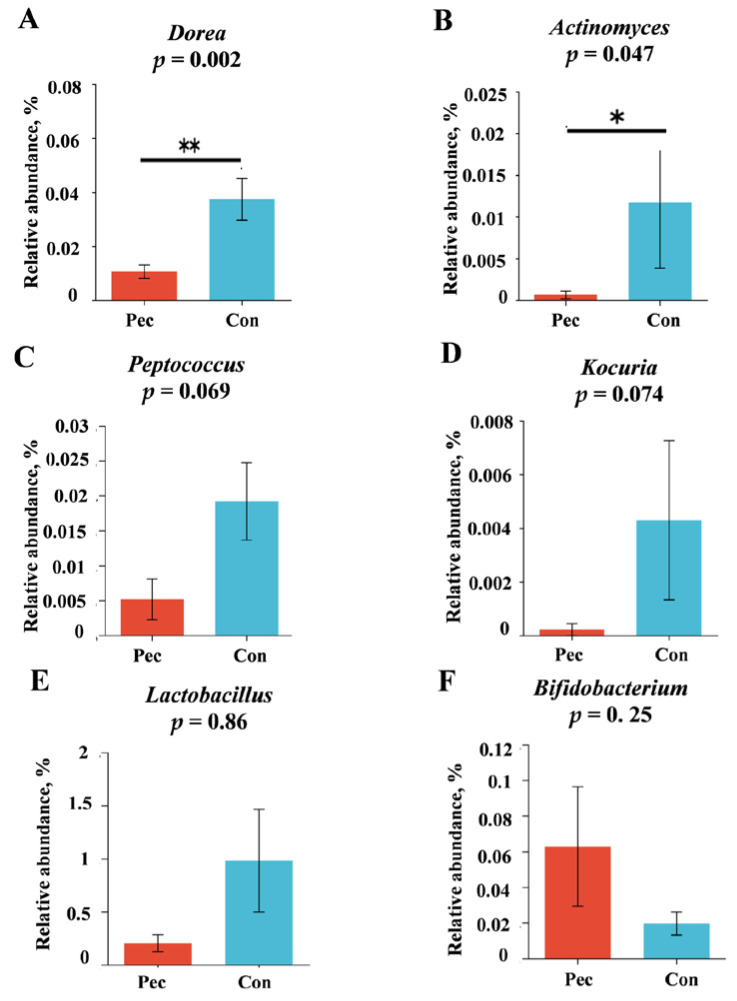
Effects in Pec and Con sows on the gut microbiota at the genus level on day 110 of gestation. (**A**) *Dorea*; (**B**) *Actinomyces*; (**C**) *Peptococcus*; (**D**) *Kocuria*; (**E**) *Lactobacillus*; (**F**) *Bifidobacterium*. Pec, pectin, *n* = 9; Con, control, *n* = 9. * indicates *p* < 0.05, ** indicates *p* < 0.01.

The relative abundance levels of the top five microbial phyla and top 10 genera are presented in Figure 3A, C. Firmicutes, Spirochaetota, Bacteriodota, Proteobacteria, and Actinobacteriota were the top five microorganisms at the phylum level. *Clostridium_sensu_stricto_1*, *Terrisporobacter*, *Romboutsia*, *Christensenellaceae_R-7_group*, *Turicibacter, Treponema*, *unclassified_f_Lachnospiraceae*, *Lachnospiraceae_xpb1014_group*, *norank_f_norank_o_Clostridia_UCG-014* , and *Sarcina* were the top 10 microorganisms at the genus level. Concretely, the changes in relative abundance at the phylum level in the Pec and Con group are presented in Figure 4. On day 110 of gestation, the relative abundance of *Firmicute* and *Bacteroidota* in the Pec group tended to be higher and lower than in the Con group, respectively (*p* = 0.077; *p* = 0.064, respectively). Compared with the Con group, the relative abundance of *Ruminococcaceae* (*p* = 0.093) and *Clostridiaceae* (*p* = 0.064) tended to increase in the Pec group, while the relative abundance of *Micrococcaceae* (*p* = 0.017) and *Coriobacteriaceae* (*p* = 0.034) at the family level tended to decrease (Figure 5). Compared with the Con group, the Pec group showed a lower relative abundance of *Dorea* (*p* = 0.002) and *Actinomyces* (*p* = 0.047) and a tendency to a higher relative abundance of *unclassified_o__Clostridiales* (*p* = 0.052) and *unclassified_o__Oscillospirales* (*p* = 0.093) at the genus level (Figure 6).

### 3.4. Plasma Biochemical Indices

The results are presented in Table 5. Compared with the Con group, the plasma concentration of TG, TC, HDL-C, TBA, and estradiol were increased in the Pec group (*p* < 0.05). These results suggested that pectin may have a certain effect on the energy metabolism of pregnant sows.

### 3.5. Plasma Inflammatory Cytokines

The results are presented in Table 6. Compared with the Con group, the plasma concentration of the pro-inflammatory cytokines IL-6, IL-1β, and TNF-α were significantly decreased in Pec sows on day 110 of gestation (*p* = 0.002, *p* < 0.01, and *p* < 0.01, respectively). The level of the pro-inflammatory cytokine IFN-γ also showed a statistical tendency to decrease (*p* < 0.10). 

### 3.6. Changes in the Levels of Intestinal Cytokines and Zonulin in Sows during Late Gestation

We also measured plasma intestinal permeability-related indicators in sows on day 110 of gestation. We found a significant reduction in the plasma zonulin level (*p* < 0.05), while the plasma LCN-2 level showed a statistical tendency to decrease (*p* < 0.10) (Table 7). 

### 3.7. Plasma SCFA Levels in Sows on Day 110 of Gestation

The levels of SCFAs are shown in Table 8. Te Pectin significantly affected the plasma SCFA levels in sows on day 110 of gestation. Compared with the Con group, the concentrations of acetic acid, propionic acid, and total SCFAs in the plasma of the sows in the Pec group were significantly increased (*p* < 0.05), and the concentration of butyric acid showed a statistical tendency to increase (*p* < 0.10).

## 4. Discussion

Gestation is an important period in the reproductive cycle of sows. In our present study, the use of pectin during gestation could increase the litter weight of newborn piglets and tended to decrease the number of mummies. Several meta-analyses have consistently demonstrated a significant association between a higher dietary fiber intake and a decreased risk of morbidity and mortality [38,39,40]. A similar phenomenon has been observed in studies conducted on pigs [18]. Previous studies indicated that the use of dietary fiber in gestational sows had some beneficial effects on sows’ production performance, such as reducing the duration of parturition [41] and increasing the feed intake of sows during lactation [42] and the litter size [43]. Unfortunately, these phenomena were not observed in our study. In this trial, we only observed that the addition of pectin during gestation in the diet of sows tended to reduce the number of mummified fetuses at delivery and increase the litter weight at birth. This difference may be due to the different types of dietary fiber used in our and other studies and the limited number of sows. However, previous studies conducted limited experiments directly including high-purity pectin fiber in sow diets during the whole gestation. We hypothesize that the differences in production performance may be due to the fiber type and the duration of its supplementation. The results of these experiments provide limited data support for the effect of pectin on the re-productive performance of sows.

In our results, the use of the soluble dietary fiber pectin during the whole gestation decreased the levels of proinflammation cytokines by improving the gut microbiota composition in the sows. Recently, a growing body of research has shown that changes in the gut microbiota during pregnancy were closely related to sow health [1,13,14,15]. Pectin fiber is a type of soluble fiber that exhibits strong fermentability, enabling it to be degraded and utilized by the gut microbiota, thereby altering the composition of the microbial communities within this region [44]. Pectin can act as a valuable carbon source for gut bacteria [45]. However, current research on pectin is mostly based on in vitro fermentation and is performed in mice. In the present study, the results of the PCoA analysis revealed differences in the gut microbiota composition between the sows in the Pec and Con groups. The present findings align with those of the study conducted by Luo et al. (2022), which reported that the use of fiber can change the microbiota in the ileum and colonic chyme in growing pigs [46]. Tian et al. (2017) reported that the consumption of pectin contributes to alterations in the gut microbiota in weaning pigs [47]. In our study, a higher abundance of Firmicutes and a lower abundance of Bacteroidota at the phyla level were observed in the Pec group compared with the Con group. This present finding aligns with prior research in a broad sense [48]. Tian et al. (2016) reported that the levels of Firmicutes in both cecal and colonic digesta were higher in rats fed with pectin-supplemented diets than in Con-fed rats. In contrast, Bacteroidetes generally decreased in relative abundance in rats fed with pectin-added diets [48]. Furthermore, some in vitro fermentation tests indicated that pectin could increase the abundance of *Ruminococcaceae* and *Clostridiaceae* [49,50]. Similarly, the abundance of *Ruminococcaceae* and *Clostridiaceae* in the Pec group was higher than in the Con group in our study. The presence of these bacteria could enhance the production of SCFAs [46,51]. In monogastric animals, pectin is degraded by fermentation by the gut microbiota to produce SCFAs [31]. Numerous recent publications have shown that SCFAs play an important role in metabolism [34,52] and immunity [35]. As a type of free fatty acids, SCFAs serve not only as a vital energy source for the body but also as crucial cell signaling molecules that actively participate in various physiological reactions within the body [53]. The inclusion of pectin was also documented to enhance the abundance of *Bifidobacterium* and *Clostridium*, as well as elevate the butyrate levels in humans in in vitro experiments [54,55]. We observed an increase in the levels of plasma acetic acid, propionic acid, total SCFAs and an increasing trend for plasma butyric concentrations in the pectin group sows. These findings suggest that pectin could alter the gut microbial community structure by increasing the abundance of some gut bacteria that increase the plasma SCFA concentration. However, the abundance of *Lactobacillus*, *Bifidobacterium*, and other beneficial bacteria in the Pec group was not changed in our study. Inconsistently, the use of pectin was able to increase the abundance of gut beneficial bacteria in rodents in previous research [48,56]. Thus, the potential of pectin to stabilize a healthy microbiome in sows as well as limit harmful bacteria should be further explored [27]. Moreover, we found a higher relative abundance of *Micrococcaceae* and *Coriobacteriaceae* in the Con sows than in the Pec sows at the family level. The *Coriobacteriaceae* family, belonging to Actinobacteria, showed increased abundance in women with high levels of serum insulin and C-peptide [57]. On the other hand, the control group exhibited a higher relative abundance at the genus level for *Dorea* and *Actinomyces*, which belong to the *Coriobacteriaceae*, compared to the pectin group. This finding is in line with the result indicating a significant prevalence of *Coriobacteriaceae* in the control group. As we know, *Actinomyces* are common pathogens [58]. The relative abundance of *Actinobacteria* increased gradually during pregnancy [59], and a high abundance of *Actinomyces* can increase the expression level of pro-inflammatory cytokines in late pregnancy, which was reported to be related to an increase in *Coriobacteriaceae* [59]. *Dorea* was positively correlated with inflammation and was reported to be highly abundant in the microbiota of patients with colitis [13]. These findings imply that pectin exhibits a certain degree of efficacy in reducing the abundance of pathogenic bacteria (such as *Dorea* and *Coriobacteriaceae*) within the gastrointestinal tract. We found that pectin did indeed increase the plasma concentrations of biochemical metabolites in sows on day 110 of gestation. These results indicate that pectin has an important effect on energy metabolism in pregnant sows. A recent study revealed that sows had symptoms of metabolic syndrome during late gestation, characterized by low levels of inflammation and metabolic disorders [1]. Therefore, the intestinal permeability and the levels of pro-inflammatory cytokines in sows were investigated during the late stage of pregnancy. The results of our study demonstrated that the use of pectin in pregnant sows could effectively mitigate intestinal permeability and reduce the plasma proinflammatory cytokine concentrations in sows after 110 days of gestation. Similarly, a previous study had indicated that DF supplementation could decrease the plasma pro-inflammatory cytokine IL-6, TNF-α, and liponnulin-2 concentrations in sows on day 110 of gestation [60]. This is consistent with previous results showing that the relative abundance of pro-inflammatory microorganisms in the pectin group decreased. Moreover, the increase in butyrate in the Pec group could regulate both the intestinal barrier and immune tolerance in animals, thereby improving the occurrence of metabolic syndrome [61,62].

In vitro and in vivo studies have shown that pectin has antioxidant [22] and anti-inflammatory effects [24] and regulates other immune activities [25]. The results of our study suggest that pectin altered the composition of the gut microbiota of sows after 110 days of gestation, leading to increased levels of SCFAs in the plasma and reduced intestinal permeability and proinflammatory markers in late pregnancy, ultimately improving sow health and reproductive performance.

## 5. Conclusions

The use of pectin during gestation could improve the production performance and health of sows by increasing the abundance of *Ruminococcaceae* and *Clostridiaceae* and decreasing the abundance of *Dorea* and *Actinomyces*. The use of pectin during gestation could increase the concentration of SCFAs and reduce intestinal permeability and cytokine levels to relieve systemic inflammation in sows. Our study provides a reference for the use of pectin in animals.

## Figures and Tables

**Table 1:** Composition and nutrient levels of the gestation diets (as-fed basis).

Item, %	Gestation		Lactation
	Con	Pec	
Corn	79.59	76.72	62.89
Dehulled soybean meal	14.0	14.0	22.13
Wheat bran	-	-	6.0
Fish meal	1.5	1.5	2.6
Soybean oil	1.5	1.5	2.0
Pectin	-	3.0	-
_L_-Lysine HCl (98%)	0.08	0.06	0.27
_D,L_-Methionine (99%)	-	-	0.13
_L_-Threonine (98.5%)	0.08	0.07	-
Limestone	1.1	1.06	0.98
Dicalcium phosphate	1.2	1.16	1.5
Sodium chloride	0.4	0.39	0.4
Choline chloride (50%)	0.15	0.14	0.15
Vitamin and mineral premix	0.4 ^1^	0.4 ^1^	0.5 ^2^
Total	100.00	100.00	100.00
Nutrient levels ^3^			
DE, Mcal/kg	3.38	3.28	3.27
NE, Mcal/kg	2.52	2.45	-
CP, %	14.05	13.81	17.50
EE, %	4.59	4.49	
SDF, %	1.72	4.65	
IDF, %	10.36	10.13	
IDF/SDF	6.02	2.18	
DF, %	12.08	14.78	
Ca, %	0.88	0.86	0.90
AP, %	0.38	0.37	0.90
SID-Lys, %	0.65	0.63	0.98
SID-Met, %	0.20	0.20	
SID-Thr, %	0.51	0.49	
SID-Trp, %	0.13	0.13	

Con, control; Pec, pectin. ^1^ Trace mineral and vitamin premix provided per kilogram of diet: Zn (as zinc sulfate), 120 mg; Cu (as copper sulfate), 20 mg; Fe (as ferrous sulfate), 120 mg; Mn (as manganese sulfate), 60 mg; I (as potassium iodide), 0.50 mg; Se (as sodium selenite), 0.30 mg; vitamin A, 10,000 IU; vitamin D_3_, 2000 IU; vitamin E, 60 IU; vitamin K, 5.0 mg; vitamin B_1_, 5.0 mg; vitamin B_2_, 10.0 mg; vitamin B_6_, 6.0 mg; vitamin B_12_, 50 μg; nicotinic acid, 40 mg; pantothenic acid, 20 mg; folic acid, 2.0 mg. ^2^ Mineral and vitamin premixes provided per kilogram of lactational diet: Fe, 120 mg; Cu, 20 mg; Mn, 30 mg; Zn, 120 mg; Se, 0.3 mg; I, 0.3 mg; vitamin A, 6000 IU; vitamin D_3_, 1200 IU; vitamin E, 50 IU; vitamin B_1_, 1.0 mg; vitamin B_2_, 3.6 mg; vitamin B_6_, 1.8 mg; vitamin B_12_, 12.5 μg; nicotinic acid, 20 mg; pantothenic acid, 12.5 mg; folic acid, 2.0 mg. ^3^ Calculated according to Chinese Feed Database (2018).

**Table 2:** Daily feed allowances of pregnant sows and average daily intake of dietary fiber.

Item	Day of Gestation (d)		
	0–30	30–90	90 Farrowing
Daily feed allowances			
Con, kg/d	2.40	2.30	2.70
Pec, kg/d	2.49	2.37	2.78

Con, control; Pec, pectin.

**Table 3:** Average feed intake and daily nutrient intake of pregnant sows.

Item	Con	Pec
ADFI, kg/d	2.41	2.49
NE, Mcal/d	6.08	6.09
CP, g/d	338.77	343.50
SDF, g/d	41.37	115.74
IDF, g/d	249.71	252.08
IDF/SDF	6.03	2.18
DF, g/d	291.08	367.82
Ca, g/d	21.27	21.29
AP, g/d	9.07	9.11
SID-Lys, g/d	15.68	15.73
SID-Met, g/d	4.92	4.99
SID-Thr, g/d	12.29	12.28
SID-Trp, g/d	3.08	3.15

Con, control; Pec, pectin; ADFI, average daily feed intake; NE, net energy; CP, crude protein; SDF, soluble dietary fiber; IDF, insoluble dietary fiber; DF, dietary fiber; Ca, calcium; AP, available phosphorus.

**Table 4:** Performance of the sows and their offspring.

Items	Treatment		*p*-Value
	Con	Pec	
Litter size at birth, No/litter	15	15	
Total born, *n*	15.09 ± 0.04	15.46 ± 0.03	0.836
Born alive, *n*	13.36 ± 0.06	13.85 ± 0.05	0.812
Stillborn, *n*	0.33 ± 0.14	0.13 ± 0.09	0.250
Mummified fetuses, *n*	2.67 ± 0.17	1.00 ± 0.29	0.099
Low-birth-weight piglet ^1^, *n*	0.74 ± 0.15	0.41 ± 0.17	0.155
Piglet weight at birth, kg	1.41 ± 0.19	1.43 ± 0.16	0.653
Litter weight at birth, kg	18.77 ± 1.21	21.85 ± 0.95	0.058
CVbw ^2^, %	21.73 ± 1.72	19.97 ± 1.45	0.442
ADFI during lactation, kg/d			
d 1–7	3.19 ± 0.32	3.50 ± 0.26	0.448
d 8–14	6.21 ± 0.32	6.02 ± 0.20	0.599
d 15–21	6.26 ± 0.37	6.25 ± 0.25	0.981
Total	5.18 ± 0.22	5.09 ± 0.13	0.711

^1^ Low birth weight piglet: Piglets with low birth weight (<1000 g). ^2^ CVbw%: the intra litter coefficient of variation. Con, control group; Pec, pectin group.

**Table 5:** Plasma biochemical indexes of sows on day 110 during gestation.

Items	Treatment		*p*-Value
	Con	Pectin	
TP, g/L	78.04 ± 1.57	85.33 ± 2.05	0.009
GLU, mmol/L	3.96 ± 0.11	3.89 ± 0.14	0.675
UREA, mmol/L	3.24 ± 0.20	3.16 ± 0.15	0.763
TC, mmol/L	131.47 ± 3.06	142.46 ± 3.39	0.070
HDL-C, mmol/L	0.45 ± 0.04	0.59 ± 0.02	0.001
LDL-C, mmol/L	0.65 ± 0.02	0.65 ± 0.03	0.939
TBA, μmol/L	15.10 ± 1.72	21.39 ± 2.85	0.082
NEFA, mmol/L	0.26 ± 0.03	0.30 ± 0.03	0.375
TG, mmol/L	0.36 ± 0.03	0.31 ± 0.02	0.228
Estradiol, pg/mL	78.04 ± 1.57	85.33 ± 2.08	0.027

Abbreviations: Con, control group; Pec, pectin group; TP, total protein; GLU, glucose; TC, total cholesterol; HDL-C, high-density lipoprotein cholesterol; LDL-C, low-density lipoprotein cholesterol; TBA, total bile acid; NEFA, non-esterified fatty acid; TG, triglyceride. Data are shown as means and SE.

**Table 6:** Plasma inflammatory cytokine levels in sows on day 110 during gestation.

Items	Treatment		*p* Value
	Con	Pec	
IL-6, pg/mL	756.212 ± 24.76	647.781 ± 19.69	0.002
IL-10, pg/mL	142.400 ± 5.047	135.371 ± 1.82	0.155
IL-1β, pg/mL	660.178 ± 16.70	562.002 ± 12.35	<0.01
TNF-α, pg/mL	200.516 ± 6.67	179.009 ± 1.80	<0.01
IFN-γ, pg/mL	35.861 ± 1.33	33.402 ± 0.74	0.097

Abbreviations: Con, control group; Pec, pectin group; IL-6, interleukin-6; IL-10, interleukin-10; TNF-α, tumor necrosis factor-α; IFN-γ, interferon-γ. Data are shown as means and SE.

**Table 7:** Plasma intestinal barrier indicators in sows on day 110 during gestation.

Items	Treatment		*p* Value
	Con	Pec	
LCN-2, pg/mL	10.458 ± 0.38	9.341 ± 0.43	0.079
Zonulin, ng/L	610.452 ± 34.15	522.237 ± 21.29	0.046
ZO-1, pg/mL	148.759 ± 8.08	143.044 ± 8.52	0.651
Occludin, pg/mL	257.742 ± 7.74	249.183 ± 10.55	0.533

Abbreviations: Con, control group; Pec, pectin group; LCN-2, lipocalin-2, ZO-1, zonula occludens protein 1. Data are shown as means and SE.

**Table 8:** Plasma SCFA concentrations in sows on day 110 during gestation.

Items	Treatment		*p* Value
	Con	Pec	
Acetate, μmol/L	424.69 ± 12.20	486.54 ± 14.59	0.004
Propionate, μmol/L	85.14 ± 16.10	166.08 ± 12.06	0.001
Butyrate, μmol/L	22.91 ± 5.97	44.24 ± 8.09	0.085
Total SCFAs, μmol/L	441.97 ± 57.09	673.87 ± 26.46	0.001

Abbreviations: Con, control group; Pec, pectin group. Data are shown as means and SE.

## Data Availability

The data are contained within the article. For more detailed data of this study, please send a request to the corresponding author.
